# Supplementary material for: Activation of the dopaminergic pathway from VTA to the medial olfactory tubercle generates odor-preference and reward
Source: eLife. 2017 Dec 18;6:e25423. doi: 10.7554/eLife.25423 (PMC5777817; doi:10.7554/eLife.25423)
Supplement: Figure 1—source data 1. [file elife-25423-fig1-data1.docx]

**Source Data for Figure 1J**

| Slice number | RV+ cells | TH+(in RV+)cells | TH+/RV+ |
| --- | --- | --- | --- |
| Animal 1#-1 | 5 | 1 | 0.454545 |
| Animal 1#-2 | 11 | 5 |  |
| Animal 1#-3 | 4 | 2 |  |
| Animal 1#-4 | 2 | 2 |  |
| Animal 2#-1 | 3 | 1 | 0.575 |
| Animal 2#-2 | 10 | 9 |  |
| Animal 2#-3 | 11 | 5 |  |
| Animal 2#-4 | 10 | 6 |  |
| Animal 2#-5 | 6 | 2 |  |
| Animal 3#-1 | 3 | 0 | 0.59375 |
| Animal 3#-2 | 10 | 7 |  |
| Animal 3#-3 | 11 | 7 |  |
| Animal 3#-4 | 8 | 5 |  |
