# Supplementary material for: Activation of the dopaminergic pathway from VTA to the medial olfactory tubercle generates odor-preference and reward
Source: eLife. 2017 Dec 18;6:e25423. doi: 10.7554/eLife.25423 (PMC5777817; doi:10.7554/eLife.25423)
Supplement: Figure 2—source data 1. [file elife-25423-fig2-data1.docx]

**Source Data for Figure 2D**

**1) Sucrose solution**

| Group | Trial | ⊿F/F |
| --- | --- | --- |
| Ctrl 1# | 1 | 0.005783 |
| Ctrl 1# | 2 | 0.004665 |
| Ctrl 1# | 3 | 0.007047 |
| Ctrl 1# | 4 | 0.002694 |
| Ctrl 1# | 5 | 0.002317 |
| Ctrl 1# | 6 | 0.002148 |
| Ctrl 2# | 1 | 0.007599 |
| Ctrl 2# | 2 | -0.003285 |
| Ctrl 2# | 3 | 0.002589 |
| Ctrl 2# | 4 | -0.006463 |
| Ctrl 2# | 5 | -0.011137 |
| Ctrl 2# | 6 | -0.006124 |
| Ctrl 2# | 7 | -0.008096 |
| Ctrl 3# | 1 | -0.001410 |
| Ctrl 3# | 2 | 0.001816 |
| Expe 1# | 1 | 0.010888 |
| Expe 1# | 2 | 0.034899 |
| Expe 2# | 1 | 0.028588 |
| Expe 2# | 2 | 0.027511 |
| Expe 2# | 3 | 0.014582 |
| Expe 2# | 4 | 0.020544 |
| Expe 2# | 5 | 0.015002 |
| Expe 3# | 1 | 0.035449 |
| Expe 3# | 2 | 0.020359 |
| Expe 3# | 3 | 0.018355 |
| Expe 3# | 4 | 0.017315 |
| Expe 3# | 5 | 0.024411 |
| Expe 4# | 1 | 0.032880 |
| Expe 4# | 2 | 0.024988 |
| Expe 4# | 3 | 0.014204 |
| Expe 4# | 4 | 0.015678 |
| Expe 4# | 5 | 0.023723 |
| Expe 5# | 1 | 0.032499 |
| Expe 5# | 2 | 0.023704 |
| Expe 5# | 3 | 0.020168 |
| Expe 5# | 4 | 0.013685 |
| Expe 5# | 5 | 0.021099 |
| Expe 6# | 1 | 0.032614 |
| Expe 6# | 2 | 0.022798 |
| Expe 6# | 3 | 0.020060 |
| Expe 6# | 4 | 0.015171 |
| Expe 6# | 5 | 0.014390 |

**2) Food pellets**

| Group | Trial | ⊿F/F |
| --- | --- | --- |
| Ctrl 1# | 1 | 0.003813 |
| Ctrl 1# | 2 | 0.003214 |
| Ctrl 1# | 3 | -0.004664 |
| Ctrl 2# | 1 | -0.009510 |
| Ctrl 2# | 2 | 0.005600 |
| Ctrl 2# | 3 | -0.010517 |
| Ctrl 2# | 4 | 0.004661 |
| Ctrl 2# | 5 | 0.003011 |
| Ctrl 3# | 1 | -0.000838 |
| Ctrl 3# | 2 | 0.001595 |
| Ctrl 3# | 3 | -0.003058 |
| Ctrl 3# | 4 | -0.001678 |
| Expe 1# | 1 | 0.010126 |
| Expe 1# | 2 | 0.008312 |
| Expe 2# | 1 | -0.010777 |
| Expe 3# | 1 | 0.008158 |
| Expe 4# | 1 | 0.011830 |
| Expe 4# | 2 | 0.011867 |
| Expe 4# | 3 | 0.018851 |
| Expe 4# | 4 | 0.019597 |
| Expe 4# | 5 | 0.016424 |
| Expe 4# | 6 | 0.005523 |
| Expe 6# | 1 | 0.010037 |
| Expe 6# | 2 | 0.011012 |
| Expe 6# | 3 | 0.006374 |

**3) Social interaction**

| Group | Trial | ⊿F/F |
| --- | --- | --- |
| Ctrl 1# | 1 | 0.002172 |
| Ctrl 1# | 2 | 0.00112 |
| Ctrl 1# | 3 | 0.000635 |
| Ctrl 1# | 4 | 0.001077 |
| Ctrl 1# | 5 | 0.000884 |
| Ctrl 1# | 6 | 0.001955 |
| Ctrl 2# | 1 | 0.001782 |
| Ctrl 2# | 2 | -0.000431 |
| Ctrl 2# | 3 | 0.000643 |
| Ctrl 2# | 4 | -0.002412 |
| Ctrl 2# | 5 | -0.001612 |
| Ctrl 2# | 6 | 0.000915 |
| Ctrl 2# | 7 | -0.001129 |
| Ctrl 2# | 8 | -0.000916 |
| Ctrl 3# | 1 | 0.001107 |
| Ctrl 3# | 2 | -0.002113 |
| Ctrl 3# | 3 | -0.001211 |
| Ctrl 3# | 4 | 0.000505 |
| Ctrl 3# | 5 | 0.002734 |
| Ctrl 3# | 6 | 0.00051 |
| Expe 1# | 1 | 0.011425203 |
| Expe 1# | 2 | 0.010545134 |
| Expe 1# | 3 | 0.009714619 |
| Expe 1# | 4 | 0.009977433 |
| Expe 1# | 5 | 0.006503882 |
| Expe 1# | 6 | 0.006445072 |
| Expe 1# | 7 | 0.008044394 |
| Expe 1# | 8 | 0.008333999 |
| Expe 1# | 9 | 0.011414887 |
| Expe 2# | 1 | 0.011039627 |
| Expe 2# | 2 | 0.006938835 |
| Expe 2# | 3 | 0.008582434 |
| Expe 2# | 4 | 0.011328491 |
| Expe 2# | 5 | 0.009981603 |
| Expe 2# | 6 | 0.008261923 |
| Expe 3# | 1 | 0.009638914 |
| Expe 3# | 2 | 0.011657676 |
| Expe 3# | 3 | 0.007034448 |
| Expe 3# | 4 | 0.00831302 |
| Expe 3# | 5 | 0.011382676 |
| Expe 3# | 6 | 0.008426808 |
| Expe 3# | 7 | 0.008612726 |
| Expe 3# | 8 | 0.011694513 |
| Expe 3# | 9 | 0.007283095 |
| Expe 3# | 10 | 0.008883923 |
| Expe 4# | 1 | 0.009815698 |
| Expe 4# | 2 | 0.006560278 |
| Expe 4# | 3 | 0.006991501 |
| Expe 4# | 4 | 0.00801762 |
| Expe 4# | 5 | 0.010908136 |
| Expe 4# | 6 | 0.007062337 |
| Expe 4# | 7 | 0.007439313 |
| Expe 4# | 8 | 0.007905723 |
| Expe 4# | 9 | 0.008036022 |
| Expe 4# | 10 | 0.009087263 |
| Expe 4# | 11 | 0.00910983 |
| Expe 4# | 12 | 0.006575624 |
| Expe 5# | 1 | 0.005522394 |
| Expe 5# | 2 | 0.008613969 |
| Expe 5# | 3 | 0.006816872 |
| Expe 5# | 4 | 0.007727164 |
| Expe 5# | 5 | 0.00808151 |
| Expe 5# | 6 | 0.008362272 |
| Expe 5# | 7 | 0.008628153 |
| Expe 5# | 8 | 0.010434136 |
| Expe 5# | 9 | 0.011420159 |
| Expe 5# | 10 | 0.009764044 |
| Expe 5# | 11 | 0.010893621 |
| Expe 5# | 12 | 0.008688157 |
| Expe 5# | 13 | 0.007262212 |
| Expe 5# | 14 | 0.010651466 |
| Expe 6# | 1 | 0.008299437 |
| Expe 6# | 2 | 0.011483244 |
| Expe 6# | 3 | 0.010437874 |
| Expe 6# | 4 | 0.006864351 |
| Expe 6# | 5 | 0.010035087 |
| Expe 6# | 6 | 0.009483748 |
| Expe 6# | 7 | 0.008774735 |
| Expe 6# | 8 | 0.011044635 |
| Expe 6# | 9 | 0.007781107 |
| Expe 6# | 10 | 0.007339208 |
| Expe 6# | 11 | 0.008082471 |
| Expe 6# | 12 | 0.008530866 |
| Expe 6# | 13 | 0.008640634 |
