# Supplementary material for: Activation of the dopaminergic pathway from VTA to the medial olfactory tubercle generates odor-preference and reward
Source: eLife. 2017 Dec 18;6:e25423. doi: 10.7554/eLife.25423 (PMC5777817; doi:10.7554/eLife.25423)
Supplement: Figure 3—source data 1. [file elife-25423-fig3-data1.docx]

**Source Data for Figure 3F**

c-Fos+ cell number of each slice recorded

| Slice recorded | OT | NAC | LSX | dSTR | PCX |
| --- | --- | --- | --- | --- | --- |
| Ctrl 1#-1 | 57 | 42 | 40 | 64 | 26 |
| Ctrl 1#-2 | 62 | 53 | 78 | 66 | 52 |
| Ctrl 1#-3 | 33 | 26 | 195 | 143 | 63 |
| Ctrl 1#-4 | 35 | 10 | 207 | 170 | 61 |
| Ctrl 1#-5 | 26 | 9 | 149 | 176 | 50 |
| Ctrl 2#-1 | 19 | 43 | 65 | 50 | 39 |
| Ctrl 2#-2 | 6 | 12 | 113 | 58 | 29 |
| Ctrl 2#-3 | 4 | 9 | 107 | 41 | 26 |
| Ctrl 2#-4 | 10 | 6 | 106 | 95 | 38 |
| Ctrl 2#-5 | 16 | - | 58 | 104 | 44 |
| Ctrl 3#-1 | 48 | 24 | 113 | 78 | 47 |
| Ctrl 3#-2 | 41 | 31 | 187 | 91 | 76 |
| Ctrl 3#-3 | 17 | 13 | 226 | 155 | 43 |
| Ctrl 3#-4 | 18 | 15 | 170 | 185 | 45 |
| Ctrl 3#-5 | 14 | - | 138 | 251 | 49 |
| Expe 1#-1 | 138 | 274 | 143 | 44 | 369 |
| Expe 1#-2 | 103 | 143 | 212 | 68 | 269 |
| Expe 1#-3 | 126 | 100 | 253 | 49 | 205 |
| Expe 1#-4 | 145 | 99 | 295 | 141 | 316 |
| Expe 1#-5 | 29 | - | 227 | 129 | 267 |
| Expe 2#-1 | 138 | 138 | 118 | 11 | 184 |
| Expe 2#-2 | 53 | 108 | 166 | 16 | 141 |
| Expe 2#-3 | 66 | 77 | 162 | 26 | 169 |
| Expe 2#-4 | 42 | 60 | 189 | 35 | 223 |
| Expe 2#-5 | 4 | - | 181 | 87 | 205 |
| Expe 3#-1 | 207 | 247 | 213 | 105 | 345 |
| Expe 3#-2 | 157 | 145 | 264 | 99 | 301 |
| Expe 3#-3 | 184 | 161 | 325 | 162 | 284 |
| Expe 3#-4 | 92 | 95 | 243 | 154 | 286 |
| Expe 3#-5 | 75 | - | 232 | 170 | 393 |

**Fig3F Statistical analysis**

| group | Chr2 | Chr2-SE | Ctrl | Ctrl-SE | Sig. (2-tailed) |
| --- | --- | --- | --- | --- | --- |
| OT | 1074.333 | 166.5148 | 650.6667 | 136.5803 | 0.0396856714992 |
| NAC | 432 | 182.081 | 575.6667 | 148.0614 | 0.006367266000551 |
| LSX | 519.6667 | 146.2486 | 135.3333 | 55.8853 | 0.07360729688802 |
| Piri | 1319 | 251.574 | 229.3333 | 32.78211 | 0.006252874602944 |
| dSTR | 549 | 102.2815 | 97.66667 | 26.32806 | 0.4950894586399 |
