# Supplementary material for: Activation of the dopaminergic pathway from VTA to the medial olfactory tubercle generates odor-preference and reward
Source: eLife. 2017 Dec 18;6:e25423. doi: 10.7554/eLife.25423 (PMC5777817; doi:10.7554/eLife.25423)
Supplement: Figure 4—source data 1. [file elife-25423-fig4-data1.docx]

**Source Data for Figure 3K**

Time percentage spent in the stimulated side of each animal

| Animal code | 0-5 min | 5-10 min | 10-15 min | 15-20 min | 20 min |
| --- | --- | --- | --- | --- | --- |
| Ctrl 1# | 58.33333 | 46.76667 | 53.16667 | 69.86667 | 57.03333 |
| Ctrl 2# | 73.8 | 52.56667 | 73.5 | 59.83333 | 64.925 |
| Ctrl 3# | 21.16667 | 43.1 | 50.5 | 62.9 | 44.41667 |
| Ctrl 4# | 50.16667 | 66.83333 | 79.1 | 71.13333 | 66.80833 |
| Ctrl 5# | 40.66667 | 38.2 | 48.73333 | 53.93333 | 45.38333 |
| Ctrl 6# | 46.2 | 51.06667 | 39.96667 | 23.6 | 40.20833 |
| Ctrl 7# | 18.76667 | 22.56667 | 32.56667 | 57.13333 | 32.75833 |
| Expe 1# | 68.06667 | 81.7 | 69.53333 | 92.6 | 77.975 |
| Expe 2# | 63 | 83.53333 | 77.7 | 74.46667 | 74.675 |
| Expe 3# | 67.73333 | 81.46667 | 70.16667 | 92.56667 | 77.98333 |
| Expe 4# | 67.53333 | 72.36667 | 75.26667 | 74.8 | 72.49167 |
| Expe 5# | 73.03333 | 81.3 | 87.33333 | 80.56667 | 80.55833 |
| Expe 6# | 64.9 | 77.6 | 72.8 | 77.76667 | 73.26667 |
| Expe 7# | 78.2 | 74.86667 | 84.7 | 77.83333 | 78.9 |

**Fig3K Statistical analysis**

| Stage | Ctrl | SE-Ctrl | ChR2 | ChR2-Ctrl | Sig. (2-tailed)（T Test） |
| --- | --- | --- | --- | --- | --- |
| 0-5 min | 44.1571 | 8.0024 | 68.9238 | 2.0970 | 0.01490402857052 |
| 5-10 min | 45.8714 | 5.5819 | 78.9762 | 1.6854 | 0.00005079068534 |
| 10-15 min | 53.9333 | 6.8864 | 76.7857 | 2.8349 | 0.006172997301927 |
| 15-20 min | 56.9143 | 6.5257 | 81.5143 | 3.1985 | 0.003289134317314 |
| Total | 50.2190 | 5.2706 | 76.5500 | 1.2521 | 0.001378047679498 |
