# Supplementary material for: Activation of the dopaminergic pathway from VTA to the medial olfactory tubercle generates odor-preference and reward
Source: eLife. 2017 Dec 18;6:e25423. doi: 10.7554/eLife.25423 (PMC5777817; doi:10.7554/eLife.25423)
Supplement: Figure 5—source data 1. [file elife-25423-fig5-data1.docx]

**Source Data for Figure 4B**

Center entries

| Animal code | Pre | L1 | L2 | L3 | Test |
| --- | --- | --- | --- | --- | --- |
| Ctrl 1# | 10 | 18 | 11 | 10 | 7 |
| Ctrl 2# | 12 | 18 | 4 | 9 | 3 |
| Ctrl 3# | 7 | 5 | 4 | 8 | 2 |
| Ctrl 4# | 29 | 16 | 14 | 11 | 12 |
| Ctrl 5# | 11 | 8 | 11 | 20 | 20 |
| Ctrl 6# | 0 | 0 | 0 | 0 | 0 |
| Ctrl 7# | 0 | 1 | 13 | 2 | 14 |
| Expe 1# | 2 | 43 | 98 | 92 | 71 |
| Expe 2# | 3 | 23 | 91 | 67 | 55 |
| Expe 3# | 2 | 27 | 87 | 141 | 91 |
| Expe 4# | 0 | 9 | 37 | 68 | 58 |
| Expe 5# | 32 | 103 | 93 | 83 | 72 |
| Expe 6# | 19 | 38 | 94 | 79 | 75 |
| Expe 7# | 35 | 53 | 52 | 71 | 46 |

**Source Data for Figure 4C**

Percentage of center duration

| Animal code | Pre | L1 | L2 | L3 | Test |
| --- | --- | --- | --- | --- | --- |
| Ctrl 1# | 2.333333 | 3.844444 | 1.666667 | 2.433333 | 0.677778 |
| Ctrl 2# | 1.688889 | 4.855556 | 1.188889 | 2.300256 | 0.333333 |
| Ctrl 3# | 2.844444 | 1.077778 | 0.855556 | 2.711111 | 0.366667 |
| Ctrl 4# | 8.7 | 1.855556 | 2.488889 | 2.633333 | 2.188889 |
| Ctrl 5# | 1.855556 | 1.344444 | 2.011111 | 10.44444 | 2.544444 |
| Ctrl 6# | 0 | 0 | 0 | 0 | 0 |
| Ctrl 7# | 0 | 0.188533 | 3.77419 | 0.08881 | 5.133333 |
| Expe 1# | 0.233333 | 13.98889 | 42.06667 | 60.55556 | 32.93333 |
| Expe 2# | 1.622222 | 5.755556 | 26.98889 | 49.17213 | 52.95556 |
| Expe 3# | 0.4 | 7.244444 | 17.8 | 30.73333 | 19.02222 |
| Expe 4# | 0 | 1.733333 | 15.65556 | 47.01111 | 25.63333 |
| Expe 5# | 6.388889 | 29.65556 | 60.08889 | 69.67778 | 43.03333 |
| Expe 6# | 4.522222 | 25.77778 | 44.83333 | 65.63333 | 27.77778 |
| Expe 7# | 6.177778 | 19.48889 | 39.97778 | 41.42222 | 35.23333 |

**Fig4B,C Statistical analysis**

| Stage | Ctrl | SE-Ctrl | ChR2 | ChR2-Ctrl | Sig. (2-tailed)（T Test） |
| --- | --- | --- | --- | --- | --- |
| Pre | 2.4889 | 1.2037 | 2.7635 | 1.0782 | 0.8623857394061 |
| T1 | 1.8540 | .7584 | 14.8063 | 4.0044 | 0.0174522074472 |
| T2 | 1.1730 | .3920 | 35.3444 | 6.0440 | 0.001294786082396 |
| T3 | 2.9318 | 1.4377 | 52.0294 | 5.2748 | 0.0000516656282637 |
| Test | 1.6063 | .7502 | 33.7984 | 4.2993 | 0.0002478521080244 |

**Duration**

**Entre times**

| Stage | CTrl | SE-CTrl | ChR2 | ChR2-CTrl | Sig. (2-tailed)（T Test） |
| --- | --- | --- | --- | --- | --- |
| Pre | 9.8571 | 3.6996 | 13.2857 | 5.7558 | 0.6253736958255 |
| T1 | 9.4286 | 2.9750 | 42.2857 | 11.4782 | 0.01692995083205 |
| T2 | 8.1429 | 2.0404 | 78.8571 | 9.1063 | 0.0001726304877879 |
| T3 | 8.5714 | 2.4675 | 85.8571 | 9.7941 | 0.000005911046232896 |
| Test | 8.2857 | 2.7664 | 66.8571 | 5.6629 | 0.0000007858092980198 |
