# Supplementary material for: Activation of the dopaminergic pathway from VTA to the medial olfactory tubercle generates odor-preference and reward
Source: eLife. 2017 Dec 18;6:e25423. doi: 10.7554/eLife.25423 (PMC5777817; doi:10.7554/eLife.25423)
Supplement: Figure 6—source data 1. [file elife-25423-fig6-data1.docx]

**Source Data for Figure 5D**

Percentage of investigation time for TMT

| Animal | Ctrl, Pre-pairing | ChR2, Pre-pairing | Ctrl, Post-pairing | ChR2, Post-pairing |
| --- | --- | --- | --- | --- |
| Expe 1# | 0.403925 | 0.355685 | 0.379863 | 0.481481 |
| Expe 2# | 0.47678 | 0.267974 | 0.385321 | 0.297101 |
| Expe 3# | 0.351097 | 0.270732 | 0.243948 | 0.313609 |
| Expe 4# | 0.379554 | 0.353846 | 0.111374 | 0.605523 |
| Expe 5# | 0.359687 | 0.309859 | 0.348066 | 0.651007 |
| Expe 6# | 0.389486 | 0.457778 | 0.294872 | 0.351955 |
| Expe 7# | 0.381468 | 0.358255 | 0.748344***** | 0.565217 |
| Expe 8# | 0.437326 | 0.365644 | 0.47234 | 0.564246 |

*** This data was excluded from final analysis.**

**Source Data for Figure 5F**

Percentage of investigation time for geraniol after antagonist administration

| Animal | Pre-pairing | D1R/D2R antagonist + post-pairing | NS + post-pairing |
| --- | --- | --- | --- |
| Expe 1# | 0.629742 | 0.44 | 0.653914 |
| Expe 2# | 0.588235 | 0.270548 | 0.861635 |
| Expe 3# | 0.377331 | 0.466019 | 0.397849 |
| Expe 4# | 0.426396 | 0.610619 | 0.809211 |
| Expe 5# | 0.226368 | 0.376 | 0.631944 |
| Expe 6# | 0.39604 | 0.333333 | 0.607053 |
