# Supplementary material for: Activation of the dopaminergic pathway from VTA to the medial olfactory tubercle generates odor-preference and reward
Source: eLife. 2017 Dec 18;6:e25423. doi: 10.7554/eLife.25423 (PMC5777817; doi:10.7554/eLife.25423)
Supplement: Figure 7—source data 1. [file elife-25423-fig7-data1.docx]

**Source Data for Figure 7G**

Percentage of investigation time for S+ or S- odor

| Animal | S+ | S- |
| --- | --- | --- |
| Ctrl 1# | 0.535032 | 0.464968 |
| Ctrl 2# | 0.648415 | 0.351585 |
| Ctrl 3# | 0.588235 | 0.411765 |
| Ctrl 4# | 0.657449 | 0.342551 |
| Ctrl 5# | 0.609507 | 0.390493 |
| Expe 1# | 0.474843 | 0.525157 |
| Expe 2# | 0.496587 | 0.503413 |
| Expe 3# | 0.556034 | 0.443966 |
| Expe 4# | 0.527322 | 0.472678 |
| Expe 5# | 0.498197 | 0.501803 |
| Expe 6# | 0.511887 | 0.488113 |

**Source Data for Figure 7H**

Accuracy of go-no-go learning for different groups

| Animal | Learned odor pair | CNO + Learned odor pair | CNO + A new odor pair |
| --- | --- | --- | --- |
| Ctrl 1# | 95% | 95% | 90% |
| Ctrl 2# | 100% | 100% | 95% |
| Ctrl 3# | 100% | 95% | 90% |
| Ctrl 4# | 95% | 90% | 95% |
| Ctrl 5# | 90% | 85% | 85% |
| Expe 1# | 100% | 100% | 65% |
| Expe 2# | 90% | 95% | 80% |
| Expe 3# | 100% | 100% | 50% |
| Expe 4# | 95% | 80% | 65% |
| Expe 5# | 85% | 80% | 80% |
| Expe 6# | 100% | 85% | 55% |
