# Supplementary material for: Activation of the dopaminergic pathway from VTA to the medial olfactory tubercle generates odor-preference and reward
Source: eLife. 2017 Dec 18;6:e25423. doi: 10.7554/eLife.25423 (PMC5777817; doi:10.7554/eLife.25423)
Supplement: Figure 8—source data 1. [file elife-25423-fig8-data1.docx]

**Source Data for Figure 8D**

Percentage of investigation time for S+ odor

| Animal | Pre-learning | Post-learning |
| --- | --- | --- |
| Ctrl 1# | 0.683761 | 0.734043 |
| Ctrl 2# | 0.497238 | 0.754286 |
| Ctrl 3# | 0.473684 | 0.619597 |
| Ctrl 4# | 0.583333 | 0.618497 |
| Ctrl 5# | 0.600000 | 0.766798 |
| Ctrl 6# | 0.307692 | 0.573333 |
| Expe 1# | 0.408482 | 0.665354 |
| Expe 2# | 0.541628 | 0.554217 |
| Expe 3# | 0.501816 | 0.574713 |
| Expe 4# | 0.603919 | 0.488584 |
| Expe 5# | 0.548555 | 0.492806 |
| Expe 6# | 0.504755 | 0.648628 |
